# Expression of *Puroindoline a* in Durum Wheat Affects Milling and Pasting Properties

**DOI:** 10.3389/fpls.2019.00482

**Published:** 2019-04-18

**Authors:** Qiong Wang, Yin Li, Fusheng Sun, Xiaoyan Li, Pandi Wang, Guangxiao Yang, Guangyuan He

**Affiliations:** ^1^The Genetic Engineering International Cooperation Base of Chinese Ministry of Science and Technology, Key Laboratory of Molecular Biophysics of Chinese Ministry of Education, College of Life Science and Technology, Huazhong University of Science and Technology (HUST), Wuhan, China; ^2^College of Life Science and Health, Wuhan University of Science and Technology, Wuhan, China; ^3^Waksman Institute of Microbiology, Rutgers, The State University of New Jersey, Piscataway, NJ, United States

**Keywords:** durum wheat, transgenic, grain hardness, kernel texture, end-use quality, milling quality, pasting property, *Puroindolines*

## Abstract

Durum wheat has limited culinary utilizations partly due to its extremely hard kernel texture. Previously, we developed transgenic durum wheat lines with expression of the wildtype *Puroindoline a* (*Pina*) and characterized PINA’s effects on kernel hardness, total flour yield and dough mixing properties in durum wheat. The medium-hard kernel texture is potentially useful for exploring culinary applications of durum wheat. In the present study, we examined the milling parameters and flour attributes of the transgenic lines, including particle size distribution, damaged starch and water binding capacity. PINA expression results in increased break and reduction flour yield but decreased shorts. PINA expression also leads to finer flour particles and decreased starch damage. Interestingly, PINA transgenic lines showed increased peak viscosity and breakdown viscosity but leave other flour pasting parameters generally unaltered. PINA transgenic lines were associated with increased small monomeric proteins, appearing to affect gluten aggregation. Our data together with several previous results highlight distinct effects of PINs on pasting properties depending on species and variety. The medium-hard kernel texture together with improved pasting parameters may be valuable for producing a broader range of end-products from durum wheat.

## Introduction

Of the global wheat production, ∼93% are contributed by allohexaploidy common wheat (*Triticum aestivum* L.; 2n = 42, AABBDD) and ∼7% by allotetraploidy durum wheat (*Triticum turgidum* ssp *durum*; 2n = 28, AABB; North Dakota Wheat Commission, 2015). The limited production and use of durum wheat are likely related to two reasons: (i) durum wheat’s extremely-hard kernels require specialized milling equipments; (ii) the milling product, semolina, has large particle size and high level of damaged starch, limited the culinary applications (Boehm et al., 2017a,b). Nevertheless, in comparison with common wheat, durum wheat is an agronomically competitive crop with tolerance of biotic and abiotic stresses and is widely grown in low-rainfall and semiarid regions (Marti and Slafer, 2014; Kaur et al., 2015).

The extremely-hard kernel texture of durum wheat (hardness index, HI > 80) is due to lack of the D genome and the hardness (*Ha*) locus, which is located at the distal end of chromosome 5DS and harbors two causal genes for kernel hardness, *Puroindoline a* and *b* (Pina and Pinb; Morris, 2002; Bhave and Morris, 2008a,b). Expression of wildtype *Pina* and *Pinb* in common wheat gives a soft kernel phenotype (HI < 40), while specific *Pina* and/or *Pinb* alleles of deletions or mutations result in hard kernel (Giroux and Morris, 1997, 1998; Beecher et al., 2002). Transgenic studies well proved that addition of PINA in the absence of starch-bound PINB, or addition of PINB in the absence of starch-bound PINA, leads to intermediate kernel hardness, demonstrating that the starch-bound PINA and PINB (friabilin) rather than total PINs’ content is the factor controlling kernel hardness (Hogg et al., 2004, 2005; Martin et al., 2006, 2007; Swan et al., 2006; Wanjugi et al., 2007).

Because of the cause-and-effect relationship between PINs and kernel hardness, efforts have been made to alter kernel texture of durum wheat to expand its limited use. The *Ha* locus has been transferred from soft-kernel common wheat into durum wheat variety Svevo using homoeologous recombination (Morris et al., 2011). The grain characteristics, milling quality and food-processing qualities of soft-kernel durum wheat germplasm have been systematically studied (Morris et al., 2015; Heinze et al., 2016; Murray et al., 2016; Quayson et al., 2016). The transferred *Ha* locus in durum wheat leads to a soft-kernel texture, favorable milling quality, low starch damage and high flour yield with variations in baking quality depending on genetic backgrounds (Boehm et al., 2017a,b). Generally, soft-kernel durum wheat in combination with favorable alleles for end-use qualities could substantially change the flour functionalities and expand the culinary function of soft durum wheat. In parallel, transgenic durum wheat lines with medium-hard kernels have been generated via *Pina* overexpression (Luo et al., 2008; Li et al., 2014). In the transgenic lines, total PINA content was higher than that of the endogenous *Pina-D1a* from common wheat cultivar Chinese Spring (CS), whereas the starch-bound PINA levels were slightly lower than that of CS possibly due to lack of PINB. Due to its higher expression level of *Pina*, we designated the transgenic lines as *Pina*-overexpressing lines in order to be coherent with previous studies. The flour quality and dough mixing properties have been studied in *Pina*-overexpressing lines and their progeny crossed with 1Ax1-expressing lines in durum wheat. PINA expression in durum wheat results in medium-hard kernel, increased flour yield, decreased dough resistance of extension, and minor effects on dough strength (Li et al., 2012, 2014).

Besides the well characterized effects of PINs on kernel hardness, flour attributes and bread-making quality in both common and durum wheat, fewer studies have investigated PINs’ impact on pasting properties. Pasting property is largely determined by starch content and composition, and the interaction of lipids and proteins with starch granules. Starch as the major determinant of pasting property is composed of two types of polymers, amylose (AMY) and amylopectin (AP), that vary in structure. The effects of AMY/AP on pasting properties of wheat flour have been illustrated via genetic or transgenic materials (Sestili et al., 2010; Shimbata et al., 2012; Zhong et al., 2017). Previously, PINs have been shown to be able to increase glycolipids (GL) and phospholipids (PL) levels in seeds, suggesting the possible ways of PINs to mediate starch-lipid interactions (Feiz et al., 2009; Kim et al., 2012). Quantitative trait loci (QTL) mapping of wheat flour pasting parameters identifies a QTL region located on 5DS, colocalized with the *Ha* loci where Puroindolines reside (Zhang Y. et al., 2009; Zhao et al., 2009). The effects of PINs on flour pasting property has been studied in near isogenic lines (NIL) of common wheat cv. Alpowa with or without the distal portion of 5DS, and in durum wheat cv. Svevo and its soft counterpart that contains the *Ha* loci (Quayson et al., 2016). The impacts of the introgression regions containing the *Ha* locus on starch swelling and RVA parameters (PV, FV, and trough, etc.) depends on species or varieties. However, it needs to emphasize that durum wheat is a different genetic material compared to common wheat because it lacks the D genome and several QTLs of pasting properties (Zhang Y. et al., 2009; Zhao et al., 2009; Deng et al., 2015; Jin et al., 2016).

Based on the above association between PINs and pasting properties, we asked: what the effects of PINA overexpression on pasting properties are in durum wheat where PINB is lacking. To address the question, we performed rapid viscosity analysis (RVA) to characterize pasting properties in *Pina*-overexpressing and control lines in the present study. Additionally, because the *Pina*-overexpressing lines we generated expand the phenotypic diversity of kernel hardness in durum, filling the gap between soft-durum and extremely-hard durum varieties, a detailed characterization of flour attributes and milling parameters is needed to explore its potential for broadening the applications of durum wheat.

## Materials and Methods

### Plant Materials

Transgenic durum wheat lines expressing *Pina* were generated by particle bombardment with plasmids pUbi-pinA and pCa-neo using cultivar Luna as donor variety (Li et al., 2014). Plasmid pUbi-pinA contains the *Pina-D1a* gene (cloned from Chinese Spring) driven by the maize Ubiquitin promoter, while plasmid pCa-neo contains the selection marker neomycin phosphotransferase II gene (*npt II*) driven by cauliflower mosaic virus 35S (CaMV 35S) promoter. The presence, expression of the transgenes *Pina* and *nptII* and the selection of *Pina* non-segregating lines from independent transgenic events were described elsewhere (Li et al., 2014). Briefly, *Pina* and *nptII* presence were determined by PCR using genomic DNA in T_3_ and T_4_ generations. *Pina* transgenic lines were self-pollinated to obtain T_4_ seeds, and the T_3_/T_4_ families from which eight random T_4_ seeds were PINA-positive were considered as the lines homozygous for *Pina*. PINA protein extraction using the Triton X-114 method and its SDS–PAGE method were reported previously (Zhang J.R. et al., 2009; Li et al., 2014). During this selection, a PINA-negative line N-2 was identified and used as the null-segregant control. The homozygosity of PINA-positive and PINA-negative lines were further confirmed in the following two seasons (2008/09 and 2009/10). Three *Pina* non-segregant lines (three events, namely PA-1, PA-6, and PA-9) and the null-segregant line N-2 were used in the present study. Protein levels of PINA have been characterized in our previous paper (Li et al., 2014), in which transgenic lines PA-1, PA-6, and PA-9 showed significantly higher levels of total PINA than the control line CS. CS is a hexaploid wheat cultivar with wildtype *Pina-D1a* and *Pinb-D1a* genes and endogenous PINA/PINB protein levels. No PINA was detected in the negative control N-2. All three transgenic lines had slightly lower levels of starch-bound PINA proteins compared to CS. The transgenic lines, however, contained significantly lowered amounts of starch-bound PINs (PINA + PINB) compared to CS due to lack of PINB. The T_7_ generation of the transgenic lines was grown in a randomized complete block design with duplicates in Wuhan (Hubei, China). The agronomic performance, molecular characterization, grain texture and other kernel characteristics of the transgenic and control lines have been reported (Li et al., 2014).

### Determination of Grain Hardness, Flour Milling, and Flour Characteristics

Grain hardness was measured using Perten Single Kernel Characterization System (SKCS) 4100 (Perten, IL, United States) on samples of 300 seeds harvested from each plot. After tempered to 16% (w/w) moisture content for 24 h (including 1 h of agitation), seed samples per genotype and plot were milled with a Chopin CD1 mill. The milling fractions were first sieved by an 800-μm sieve followed by a 160-μm sieve after milling. The milling fraction held by the 800-μm sieve was called large brans, while the milling fraction that can pass the 800-μm sieve but not the 160-μm sieve was middlings. The milling fraction passed both sieves was break flour. The middlings were milled once again followed by passing the two sieves. After the second milling and sieving process, the milling fraction that passed both sieves was reduction flour, while the fraction hold by the 160-μm sieve were shorts. Straight-grade flour yield is calculated from the total amounts of break and reduction flour divided by amounts of all millstream (bran, shorts, reduction and break flour). Flour characteristics were obtained by near-infrared reflectance spectroscopy.

### Determination of PINA Expression Level

Seed protein was extracted from wheat flour first by 50% propan-1-ol (v/v) extraction according to Tosi et al. (2005; the isopropanol-soluble fraction) followed by a loading buffer extraction of the precipitant according to Liu et al. (2007; the isopropanol-insoluble fraction). Both of the extracts were separated by SDS–PAGE with 10% separating gels. PINA protein level was detected using Western blotting and the PINA-specific antibody with a dilution of 1:3000 (Li et al., 2014).

### Determination of Gluten Content and Index

Wet gluten was washed from straight-grade flour by using a Perten 2200 Glutomatic System (Perten Instruments AB, Huddinge, Sweden), following by the AACC 38-12-02 method (AACC, 2000). Both the wet gluten forced through on the sieve or remaining on the sieve were weighed after centrifugation. Next, the samples were dried under standardized conditions and weighed. Wet and dry gluten contents were expressed as percentage of the sample, with the ratio of the wet gluten remaining on the sieve to the total wet gluten expressed as gluten index.

### Determination of Damaged Starch and Water Binding Capacity (WBC)

Starch damage was determined in duplicate by the amperometric method with SDmatic from Chopin Technologies (Medcalf and Gilles, 1965). Straight-grade flour sample (1 g) was put into the small bucket with 120 ml the solvent (3 g boric acid, 3 g potassium iodine, 120 ml purified water and one drop of 0.1 M sodium thiosulfate) added in reaction cup. After the cup warmed up to 35°C, the flour sample were automatically transferred into it. Results reported in absorbed iodine (AI%) calculated were expressed in UCD unit (Unité Chopin Dubois).

For water binding capacity, a portion of 0.25 g of straight-grade flour was weighed in a micro-centrifuge tube where 1 mL water was added. After vibration for 5 min followed by resting for 30 min at 25°C, the flour-water mixture was centrifuged at 2000 *g* for 10 min. The supernatant was discarded, and the retained residue was weighed and expressed as the weight of flour plus the water absorbed (AACC, 2000).

### Particle Size Distribution in Suspensions of Four

Particle size distribution in suspensions of straight-grade flour was evaluated by Malvern laser particle size analyzer (Mastersizer, 2000, Malvern, United Kingdom) with a polydisperse analysis mode and a 300 mm lens. Dry samples were dispersed in isopropyl alcohol in the circulation unit to attain an obscuration of 15–20%. The sample was first mechanically agitated to be circulated in the equipment, ultrasound for 1 min to dissolve flour clots, and then measured with 2 min interval. Size distribution was determined from duplicated samples with four measurements per sample.

### Determination of Pasting Properties by Rapid Visco-Analysis

The pasting properties were determined using straight-grade flour with a Rapid Visco Analyser (RVA-4, Newport Scientific, Australia; Collar, 2003). A portion of 3.5 g flour and 25 g water were mixed in an aluminum cylindrical bowl. The following temperature variation program was applied: heating from 25 to 95°C for 6 min, holding for 3 min at 95°C and then decreased to 25°C for 7 min.

### SE-HPLC Analysis

The straight-grade flour was dissolved in 1 mL of 0.05 M sodium phosphate buffer (pH 6.9) containing 0.5% (w/v) SDS. Then the mixed system was sonicated for 15 s and centrifuged for 10 min at 13,000 *g*. The supernatant was filtered through a 0.22 μm PVDF membrane and fractionated by using a Waters HPLC system with size exclusion column according to Tosi et al. (2005). Four fractions were used to analyze the chromatographic profiles, F1 (large-sized polymers), F2 (medium-sized polymers), F3 (oligomers glutenin) and F4 (monomeric glutenin and other small non-gluten proteins; Tosi et al., 2005; Ma et al., 2013).

### Statistics Analysis

Data were analyzed using one-way analysis of variance (ANOVA) and significant differences between lines were determined using the least significant difference pairwise comparison and displayed by letters (*p* < 0.05).

## Results and Discussion

### Effects of *Pina* Expression on Grain Hardness, Flour Characteristics, Milling Attributes and Flour Particle Size in Durum Wheat

Three *Pina*-overexpressing lines of durum wheat (PA-1, PA-6, and PA-9) and a null-segregant line (N-2) had been generated previously, with the expression of PINA and kernel hardness phenotypes characterized (Li et al., 2014). PINA overexpression in the transgenic lines was confirmed when compared with the endogenous PINA in CS detected by western blotting (Supplementary Figure S1). Here, we focused on studying the effect of transgenic PINA on milling fractions. *Pina* expression led to a medium-hard kernel texture (HI ranged from ∼56 to ∼64), while the null-segregant line and non-transgenic Luna had extremely hard kernel (HI > 95) (Table 1). Consistent with the changes in kernel texture, the *Pina*-overexpressing lines differed from the control lines in milling quality. In detail, *Pina*-overexpressing lines had significantly increased break and reduction flour but decreased shorts compared to control line N-2 and non-transgenic cv. Luna (Table 1). This resulted in higher straight-grade flour yield (SFY) in transgenic lines, increased from ∼45% to over 60%. The SFY range is comparable to those reported in durum wheat cv. Svevo and soft Svevo (∼50 to ∼70%) that has been created by homoeologous recombination (Murray et al., 2016). However, the distribution of mill fractions is distinct between the previous and present studies, probably because of the different milling systems used in the two studies. The soft-kernel Svevo had markedly increased break flour yield but decreased reduction flour yield and shorts compared to the *Pina*-overexpressing lines (Table 1). In the previous study, a modified Quadrumat Senior mill was used for Svevo and soft Svevo, in which the flour samples passed through three rolls gaps established by four break rolls followed by sifting through 500 and 150 μm sieves. Here, a Chopin CD1 mill was used with sifting through 800 and 180 μm sieves. Therefore, smaller fractions were considered as bran, and the total percentages of bran and shorts were generally comparable to those of the varieties tested previously, including common wheat cv. Xerpha and Expresso. Additionally, being consistent with the fact that *Pina*-overexpressing lines have an intermediate phenotype of hardness, these transgenic lines appear to be intermediate in SFY compared with those of soft-durum lines (∼60% SFY for *Pina*-overexpressing lines versus ∼70% SFY for soft-durum lines).

**Table 1:** Grain hardness, milling attributes, flour characteristics, and color parameters of the transgenic and control lines.

	Line				
Parameters	PA-1^1^	PA-6^1^	PA-9^1^	N-2^1^	Luna^1^
Transgene	*Pina*	*Pina*	*Pina*	None	None
Grain Hardness^2^	63.6 ± 2.0b	55.8 ± 4.4b	60.4 ± 4.4b	95.1 ± 4.4a	98.7 ± 2.3a
Milling Attributes^3^					
Bran (g)	13.8 ± 0.6a	13.8 ± 0.8a	14.1 ± 0.1a	8.4 ± 0.4b	9.2 ± 0.2b
Break flour (g)	18.4 ± 0.4a	18.2 ± 0.9a	18.5 ± 0.4a	10.5 ± 0.3b	9.5 ± 1.3b
Shorts (g)	23.6 ± 0.2b	24.2 ± 1.0b	24.1 ± 0.4b	43.4 ± 1.8a	45.9 ± 4.0a
Reduction flour (g)	41.3 ± 0.8a	41.0 ± 0.7a	41.0 ± 0.7a	36.1 ± 0.5b	34.0 ± 2.3b
Total product (g)	97.1 ± 0.0b	97.3 ± 0.0b	97.7 ± 0.2b	98.4 ± 0.6a	98.5 ± 0.2a
BFY (%)	19.0 ± 0.4a	18.7 ± 0.9a	18.9 ± 0.4a	10.7 ± 0.4b	9.7 ± 1.3b
SFY (%)	61.5 ± 0.5a	60.9 ± 0.2a	60.9 ± 0.4a	47.4 ± 1.1b	44.1 ± 3.8b
Flour Characteristics^4^					
Protein content (%)	12.8 ± 0.0b	11.7 ± 0.1b	12.4 ± 01b	14.6 ± 0.1a	14.4 ± 0.1a
Water Content (%)	13.1 ± 0.0a	13.2 ± 0.1a	13.1 ± 0.1a	12.7 ± 0.1b	12.7 ± 0.0b
Ash Content (%)	0.7 ± 0.0b	0.7 ± 0.0b	0.7 ± 0.0b	0.8 ± 0.1a	0.9 ± 0.1a
Gluten Content (%)	29.2 ± 0.2b	26.9 ± 0.4b	28.7 ± 0.3b	31.9 ± 0.2a	30.9 ± 0.1a

*^1^Means with the same letter are not significantly different (P > 0.05). All data are presented as mean ± SEM. ^2^Measured by single kernel characterization system (SKCS) from 300 seeds per line and plot. ^3^Measured by Chopin CD1 mill (n = 4); BFY = break flour yield; SFY = straight-grade flour yield. ^4^Measured by near-infrared reflectance spectroscopy (NIRS) method.*

The particle size distribution of straight-grade flour was compared between the *Pina*-overexpressing and control lines. *Pina*-expressing lines had smaller particle size compared to those of the control lines (Table 2 and Supplementary Figure S2). For instance, the mean diameter (D32) of the *Pina*-expressing lines decreased significantly. It is worthy to mention that the difference in straight-grade flour particle size may be quite a minor effect of *Pina*-overexpression compared to the difference in mill fractions between *Pina*-overexpressing and control lines.

**Table 2:** Flour particle diameters of the transgenic and control lines.

	Line				
Parameters	PA-1^1^	PA-6^1^	PA-9^1^	N-2^1^	Luna^1^
Transgene	*Pina*	*Pina*	*Pina*	None	None
SSA (mˆ2/kg)^2^	205.0 ± 0.3a	206.0 ± 0.1a	209.0 ± 1.3a	178.1 ± 0.8b	177.7 ± 0.4b
D21 (μm)^3^	1.9 ± 0.0b	1.9 ± 0.1b	1.8 ± 0.1b	2.3 ± 0.0a	2.2 ± 0.0a
D32 (μm)^4^	9.4 ± 0.0b	9.5 ± 0.1b	9.3 ± 0.1b	10.9 ± 0.1a	10.9 ± 0.0a
D43 (μm)^5^	32.5 ± 1.0b	31.6 ± 0.0b	32.6 ± 1.6b	33.2 ± 1.2a	33.9 ± 0.1a
D10 (μm)^6^	4.2 ± 0.0b	4.3 ± 0.1b	4.2 ± 0.1b	5.1 ± 0.0a	5.1 ± 0.0a
D50 (μm)^7^	25.0 ± 0.4b	24.8 ± 0.1b	24.8 ± 1.0b	26.2 ± 0.1a	26.6 ± 0.1a
D90 (μm)^8^	71.8 ± 3.5b	70.9 ± 4.9b	72.4 ± 4.4a	71.6 ± 0.3b	73.2 ± 0.2a

*^1^Values within the same column followed by the same letter are not significantly different (P < 0.05). ^2^SSA: specific surface area. ^3^D21: Length mean diameter. ^4^D32: Surface Weighted Mean. ^5^D43: Volume Weighted Mean. ^6^D10: the diameter where 10% of the population lies below the D10. ^7^D50 (the median): the diameter where half of the population lies below the D50. ^8^D90: the diameter where 90% of the distribution lies below the D90.*

**FIGURE 1:**
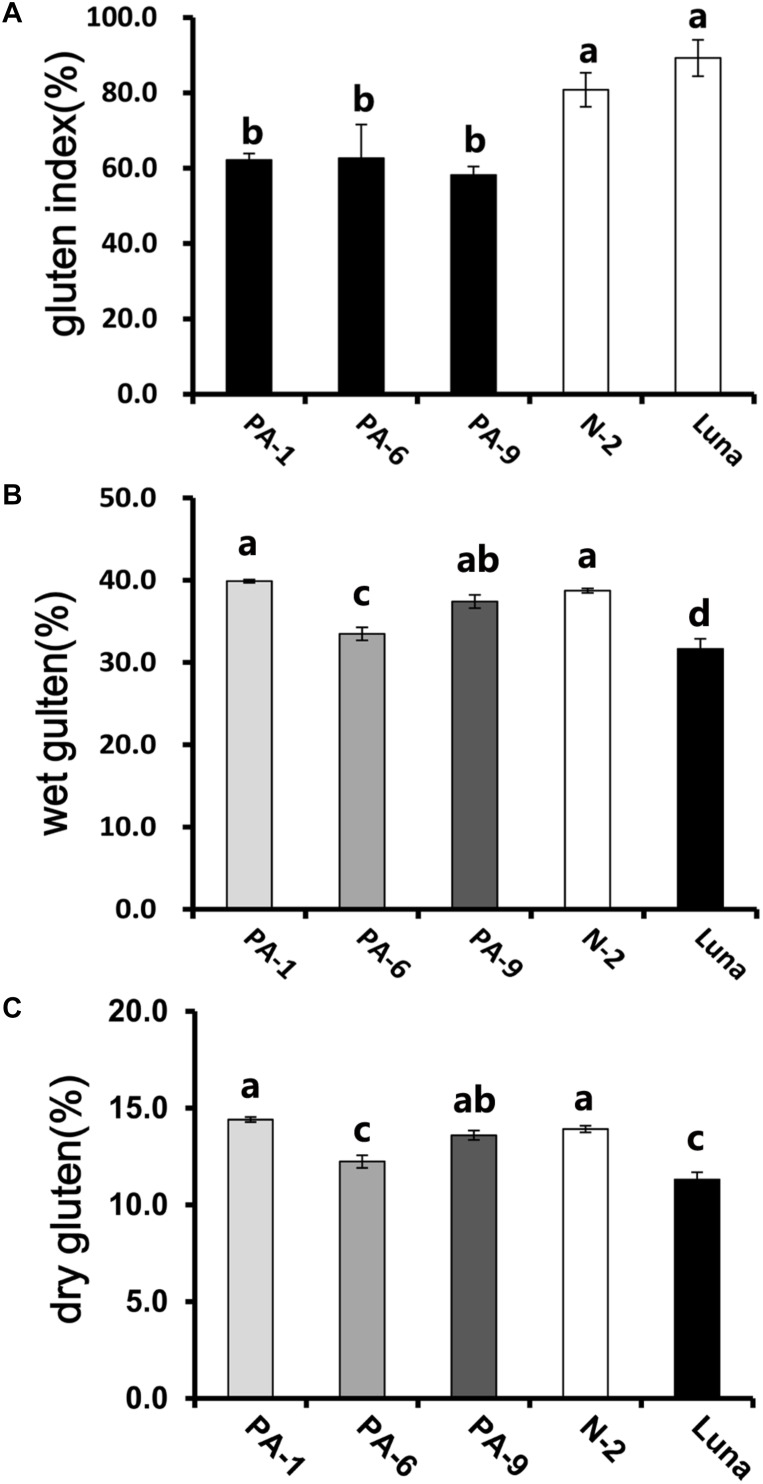
Determination of gluten index of transgenic and control lines. **(A)** The wet gluten **(B)** dry gluten content, and **(C)** gluten index of transgenic and control lines. Data are given as mean ± SEM, calculated from three replicates. Values within the column followed by different letters indicate significant difference (*P* < 0.05).

In addition, the *Pina*-expressing lines were associated with slightly increased flour water content and decreased protein content (Table 1). In our previous study, flour protein contents of these lines were slightly decreased (not significant; Li et al., 2014). In accordance with the decrease in flour protein content, the transgenic lines had significantly lowered gluten content and gluten index compared to the control lines (Figure 1). Similarly, an association of the presence of endogenous PINs with flour protein content have been observed in near isogenic lines of both common and durum wheat (soft Alpowa versus hard Alpowa for common wheat; Svevo versus soft Svevo for durum wheat; Hogg et al., 2005; Quayson et al., 2016). However, it was unable to exclude the possibility that the observed differences in protein content were due to environment and/or genotype × environment effects, as evidenced by another previous study (Boehm et al., 2017a). The result presented herein, together with previous data, suggests the necessity to investigate the effects of PINA and/or PINB expression on flour protein content under robust and rigorous field experimental conditions.

### Effects of *Pina* Expression on Damaged Starch and Water-Binding Capacity in Durum Wheat

**FIGURE 2:**
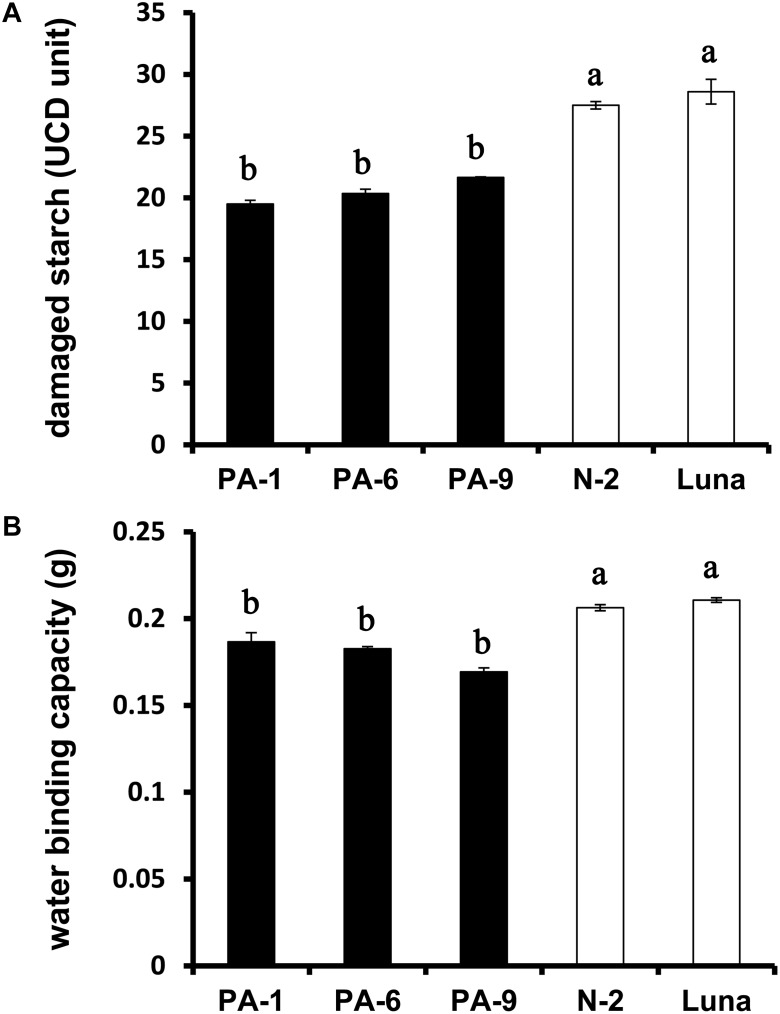
Determination of damaged starch and water-binding capacity of the transgenic and control lines. **(A)** Damaged starch content and **(B)** water-binding capacity of wheat flours. Data are provided as mean ± SEM. Damaged starch content and water binding capacity were determined from duplicated samples with triplicate measurements. Values within the column followed by different letters indicate significant difference (*P* < 0.05).

The relationships between grain hardness, starch damage and flour water absorption were studied previously (Peng et al., 1999; León et al., 2006). Generally, hard wheat grains have stronger adhesion between starch granule surface and protein matrix, and require more energy to produce fine flour, thus leading to increased starch damage after milling (Greenwell and Schofield, 1988; Yu et al., 2015). The starch damage was decreased from ∼29% for N-2 and Luna to ∼20% for the *Pina*-overexpressing lines (Figure 2A), consistent with the lowered kernel hardness in these lines. Moreover, PINA overexpression was related to decreased flour water binding capacity (Figure 2B), reflecting the positive correlation between damaged starch and flour water absorption.

### Overexpression of *Pina* Affects Flour Pasting Properties in Durum Wheat

**FIGURE 3:**
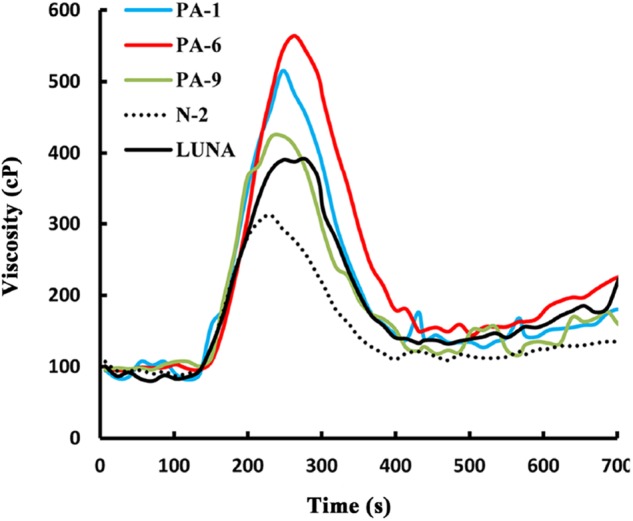
RVA pasting profile of flour from transgenic lines and control lines.

Previously, we showed that transgenic PINA expression in durum wheat led to an intermediate kernel hardness and increased flour yield (Li et al., 2014). In another study, we investigated the effects of PINA expression in durum wheat on dough mixing property resulted in decreased dough resistance of extension, and minor effects on dough strength (Li et al., 2012). Pasting parameters are a set of important traits that affect several food-processing quality, such as noodle-making quality (Zi et al., 2019) and the quality of baking powder biscuits (Ma and Baik, 2018). In the present study, the impacts of *Pina* expression on flour pasting property in durum wheat were analyzed by RVA (Figure 3 and Supplementary Figure S3) and are shown in Table 3. There was no difference in peak time and pasting temperature between the *Pina*-overexpressing and control lines. Interestingly, *Pina*-overexpressing lines presented significantly higher peak viscosity and breakdown viscosity values than the control lines, indicating greater swelling capacity with the overexpression of PINA. Since both transgenic and control lines had similar holding strength (trough), the higher breakdown viscosity values in *Pina*-overexpressing lines could be explained by their higher peak viscosity compared to the control lines (Figure 3). Moreover, the trough and final viscosity were not different between the *Pina*-overexpressing and control lines. According to previous studies, noodles especially from Asian areas require high peak and breakdown viscosity. Pasting temperature and swelling power of starch are also positively correlated to good noodle-making quality (Zi et al., 2019). Studies on white salted noodle and frozen noodle show that swelling power and stability positively contributes to the noodle quality (Yun et al., 1996; Yue et al., 2017). RVA peak viscosity was positively associated with biscuit quality, while negative correlation was detected for damaged starch (Ma and Baik, 2018). Based on food chemistry research, the modification of RVA parameters and other flour attributes could be favorable for improving the quality of certain types of end-products.

**Table 3:** RVA properties of the transgenic and control lines.

	Line				
Parameters	PA-1	PA-6	PA-9	N-2	Luna
Transgene	*Pina*	*Pina*	*Pina*	None	None
Peak Viscosity	510.5 ± 4.5b	568.0 ± 4.0a	448.5 ± 0.5c	446.5 ± 1.5c	393.0 ± 16.0d
Setback	83.5 ± 4.5a	96.5 ± 6.5a	40.0 ± 4.0b	73.5 ± 3.5ab	47.0 ± 1.0b
Breakdown	381.0 ± 7.0b	421.0 ± 7.0a	324.0 ± 5.0c	304.0 ± 0.0d	264.0 ± 6.0e
Trough	129.5 ± 2.5b	147.0 ± 3.0a	124.5 ± 5.5b	142.5 ± 1.5a	129.0 ± 10.0b
Final Viscosity	213.0 ± 7.0b	243.5 ± 3.5a	164.5 ± 9.5c	216.0 ± 2.0b	176.0 ± 11.0c
Peak Time (min)	4.2 ± 0.0	4.4 ± 0.1	4.1 ± 0.1	4.4 ± 0.0	4.3 ± 0.0
Pasting Temp (°C)	68.3 ± 1.2	69.8 ± 1.3	69.0 ± 0.4	61.1 ± 10.0	67.8 ± 4.2

*Values within the same column followed by the same letter are not significantly different (P > 0.05). All data are presented as mean ± SEM.*

Interestingly, different effects of PINA and/or PINB on RVA parameters were reported previously. In *T. aestivum*, comparison of RVA results between cv. Alpowa and its near isogenic hard Alpowa suggests PINs lead to decreased peak viscosity, trough and final viscosity. By contrast, in durum wheat, comparison between Svevo and soft Svevo shows that PINs increased peak viscosity, breakdown and setback values (Quayson et al., 2016). Moreover, comparison of RVA parameters between transgenic rice with expression of PINB and its control line demonstrates that PINB results in lowered peak viscosity, breakdown and final viscosity (Wada et al., 2010). First, the effects of PINA and/or PINB on pasting properties reported in multiple studies are unlikely to be determined by starch contents, as the plant materials used were transgenic lines and their controls, or near isogenic lines. In our case, although AMY content and amylose/amylopectin ratio were not measured, these factors seem unlikely to play major roles in the detected changes of pasting properties since the transgenic and control lines share the same genetic background (Luna). Second, the effects of PINs varied in different RVA parameters, depending on varieties, species, or the type of cereal kernels used. The mechanism underlying the differential effects of PINs on pasting properties remains unclear. Since the kernel hardness and starch damage were apparently unable to fully explain PINs’ distinct effects on RVA parameters, it may be hypothesized that potential starch-lipid or starch-protein interactions mediated by PINs b might play roles in affecting pasting parameters. It is yet to be disentangled the direct effects of PINA and/or PINB on RVA parameters through possible starch interactions from indirect effects of kernel hardness or starch damage. In summary, further investigations on the effects of PINs on pasting property using transgenic plants in various durum backgrounds are required.

### Effect of *Pina* Expression on Gluten Aggregation in Durum Wheat

**FIGURE 4:**
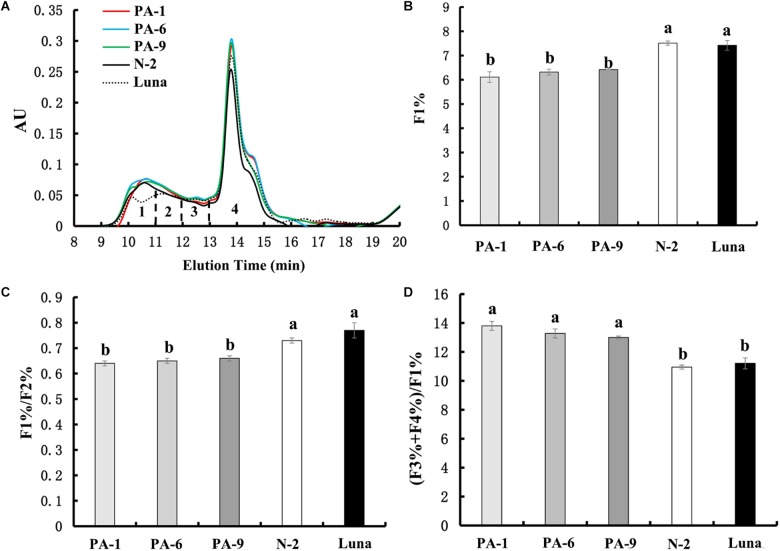
Effect of PINA on the formation of gluten aggregates. **(A)** SE-HPLC analyses of flour samples from the transgenic and control lines. **(B–D)** Comparisons of the three SE-HPLC parameters F1%, F1/F2%, and (F3+F4%)/F1% between transgenic and control lines, which are related to gluten strength and baking quality. Data are given as mean ± SEM from three replicates. Different letters on the columns within the same SE-HPLC parameters indicate significant difference (*P* < 0.05).

Recently, PINs have been shown to potentially interact with gluten proteins through hydrophobic interactions, suggesting additional mechanistic possibility for the PINs’ effects on kernel hardness (Quayson et al., 2018). Our above results of pasting properties also suggest not to exclude possible explanation related to PIN-gluten interactions. To determine whether PINA-overexpression could be associated with gluten proteins, we investigated the size distribution of gluten aggregates extracted from the transgenic and control lines (Figure 4). Four fractions can be used to analyze the chromatographic profiles, F1 (large-sized polymers), F2 (medium-sized polymers), F3 (oligomers glutenin), and F4 (monomeric glutenin and other small non-gluten proteins; Tosi et al., 2005; Ma et al., 2013). PINA overexpression increased the amounts of small monomeric proteins (peak#4 in Figure 4A) and consequently altered the relative amounts of several peaks, such as F1%, F1/F2%, and (F3+F4%)/F1% (Figure 4), which are related to dough viscoelasticity (Ma et al., 2013). Gluten aggregation parameters, such as F1%, F1/F2%, and (F3+F4%)/F1%, have strong correlations with dough quality parameters (Ma et al., 2013; Li et al., 2017). Therefore, these data show overexpression of PINA was associated with altered gluten aggregation patterns. Still, further investigations are needed to disentangle the direct impacts of PIN-gluten interaction and indirect effects of kernel texture (caused by PINs) dough property and food-processing quality.

### Advantages and Disadvantages of Durum Wheat Lines With Modified Kernel Texture

Recently, soft-kernel texture was first achieved in durum wheat cultivar Svevo and then was introgressed into a series of durum varieties. Systematic investigations on these soft-durum wheat lines show several traits improvement compared to traditional durum wheat, including soft kernel texture, less damaged starch, smaller flour particle size. The transgenic lines described here have several advantages. First, they can achieve a medium-hard kernel texture through PINA expression, together with intermediate levels of starch damage, flour particle size and distinct millstream patterns compared with soft- and extremely hard- durum wheat. These intermediate phenotypes may not be fulfilled by recombination of the *Ha* locus, since three closely-linked genes, *Pina*, *Pinb*, and *Grain Softness Protein 1* (Gsp-1), are located in this locus and *Pins* play major roles in kernel hardness and *Gsp-1* has minor effects (Wilkinson et al., 2017). The transgenic lines with these intermediate phenotypes are valuable for a broader range of end-products with enhanced quality. Stacking of genes or alleles favorable for other agronomical traits can further expand potential utilization of durum wheat, as exemplified by the stacking of HMW-GS 1Ax1 with PINA (Li et al., 2012). Third, the transgenic lines herein remind the benefits of transgenic approach that the time, location and expression level of gene of interest can be manipulated (e.g., through tissue-specific promotors). In gene function study, the benefits could also provide opportunities to dissect the direct functional consequences of PIN-gluten interaction and the indirect influence of kernel texture on food-processing qualities. However, disadvantages of the transgenic lines do exist. They are subject to restricted regulation for transgenic organism and therefore may possibly hamper their utilization in breeding programs. Utilization of the present transgenic lines could also be limited due to the non-specific expression of *Pina*. Nevertheless, it is time to unleash the kernel-hardness limitation to expand the utilization of durum wheat.

## Conclusion

The soft-durum wheat has been exemplified as a paradigm shift in terms of the grain and food processing qualities of traditional durum wheat cultivars (Boehm et al., 2017a,b). In parallel, the transgenic lines with overexpression of PINA have been generated and characterized (Li et al., 2014). Besides the medium-hard kernel texture and increased flour yield resulted from PINA overexpression that is confirmed here, detailed investigations on flour and milling attributes show that PINA overexpression increases flour yield, lower starch damage and, therefore, affects flour particle size and water absorption in durum wheat. PINA overexpression leads to elevated break and reduction flour but decreased shorts. For pasting properties, PINA overexpression is associated with increased peak viscosity, but the trough, setback and final viscosities are generally unchanged. Increased small monomeric gluten proteins was observed in PINA overexpression lines, indicating potential PINA-gluten interactions. The modified flour quality parameters associated the transgenic lines may be useful for generating broader range of end-products of durum wheat.

## Author Contributions

YL, GY, and GH designed the experiments. QW, YL, FS, XL, and PW performed the experiments. QW and YL analyzed the data. QW, YL, and GH wrote the manuscript. GY edited the manuscript. All authors read and approved the final manuscript.

## Conflict of Interest Statement

The authors declare that the research was conducted in the absence of any commercial or financial relationships that could be construed as a potential conflict of interest.
