# Supplementary material for: Expression of Puroindoline a in Durum Wheat Affects Milling and Pasting Properties
Source: Front Plant Sci. 2019 Apr 18;10:482. doi: 10.3389/fpls.2019.00482 (PMC6482235; doi:10.3389/fpls.2019.00482)
Supplement: Supplementary file 1 [file Image_1.pdf]

Supplementary Material

Supplementary Figures

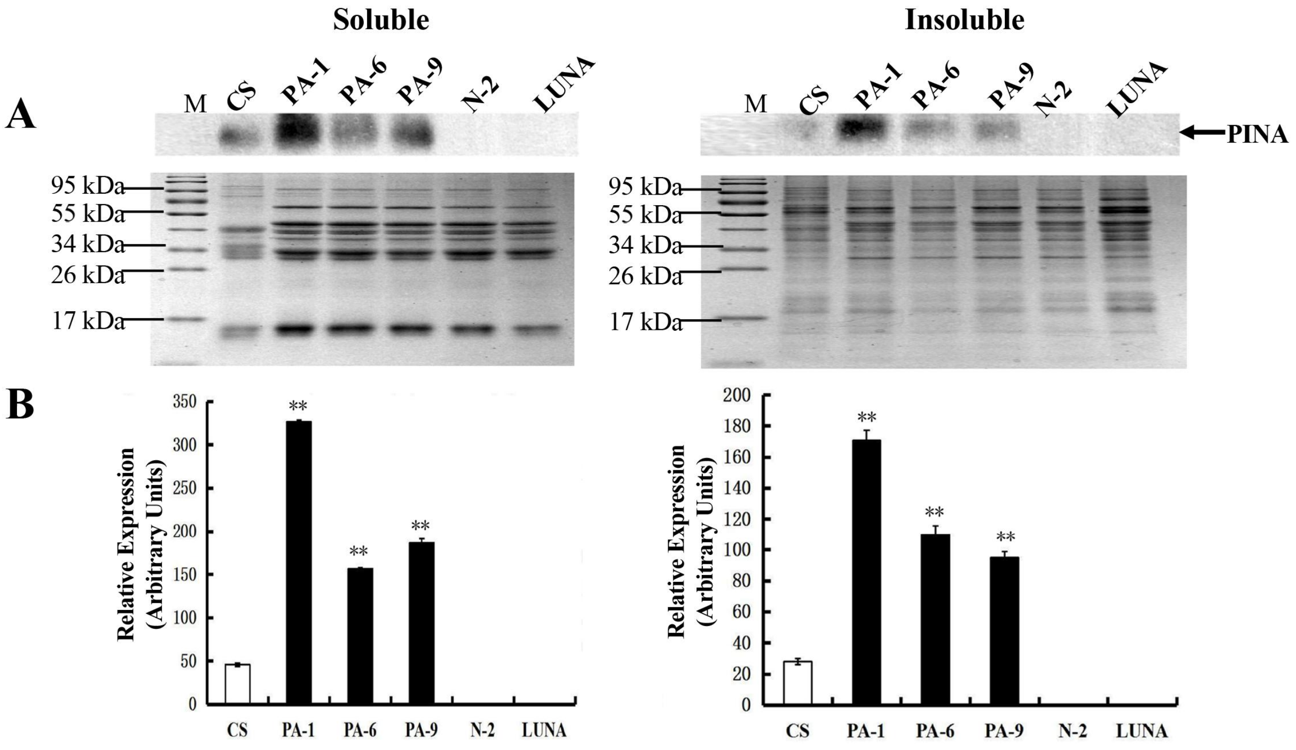

Figure S1. SDS-PAGE and Western blotting analyses of PINA distribution in total protein of transgenic and control lines. (A) Western blotting and SDS-PAGE of PINA distribution in soluble and insoluble gluten protein fractions extracted from flour. (B) Densitometry quantification of western blotting results of PINA distribution in gluten protein fractions extracted from flour. ** show the comparisons between all transgenic lines and positive controls (CS). (**$P < 0.01$). Significant difference of PINA expression determined by Western blotting was measured by Student's t test ($P < 0.01$).

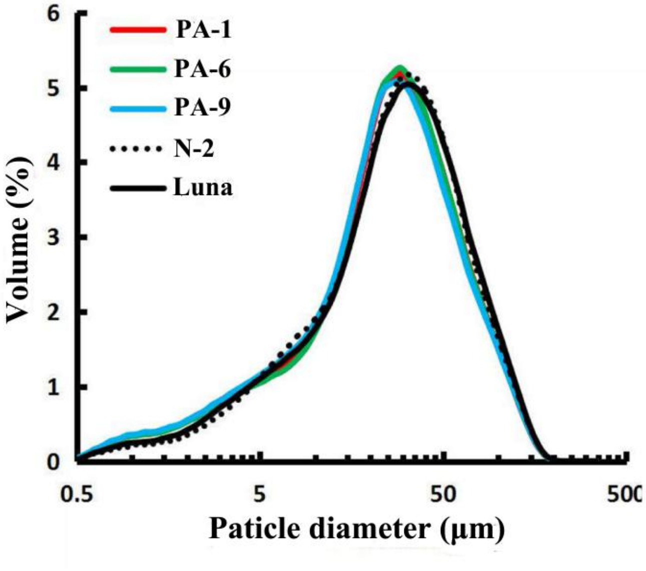

Figure S2. The size distribution profile of flour particles from transgenic lines and control lines.

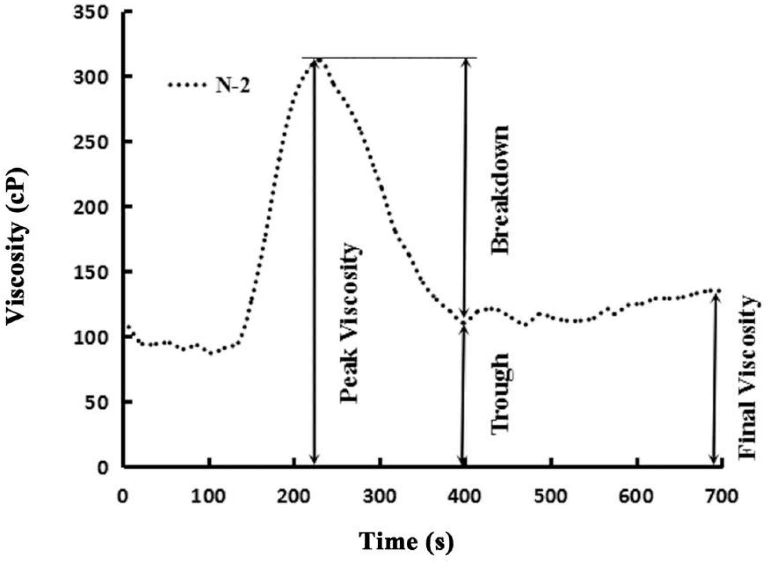

Figure S3. Schematic diagram of flour pasting property parameters measured by RVA. The most important parameters include peak viscosity, breakdown, trough viscosity, final viscosity and setback. Particularly, setback is measured as the difference between final viscosity and trough viscosity, which can be shown as setback = final viscosity-trough viscosity.

Figure S4, S5, S6 and S7 are the raw images of gel blots/electrophoresis which were used in Figure S1.

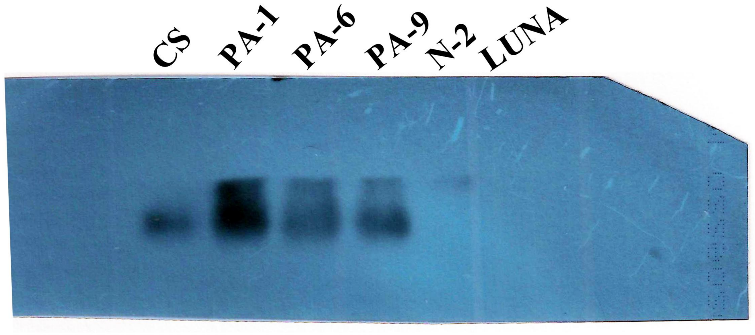

Figure S4. Original western blotting result of PINA distribution in soluble gluten protein fractions (Which appeared in Figure S1 A upper panel (Left).

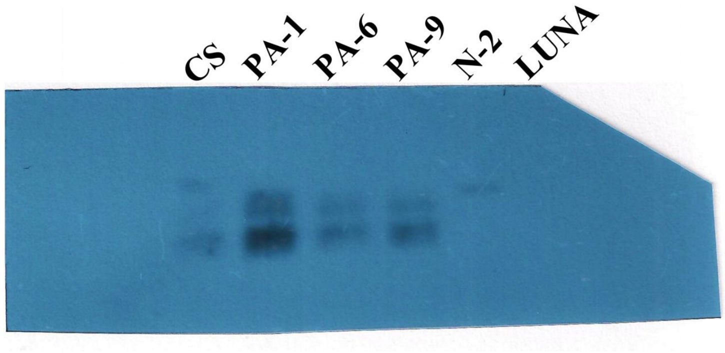

Figure S5. Original western blotting of PINA distribution in insoluble gluten protein fractions (Which appeared in Figure S1 A upper panel (Right).

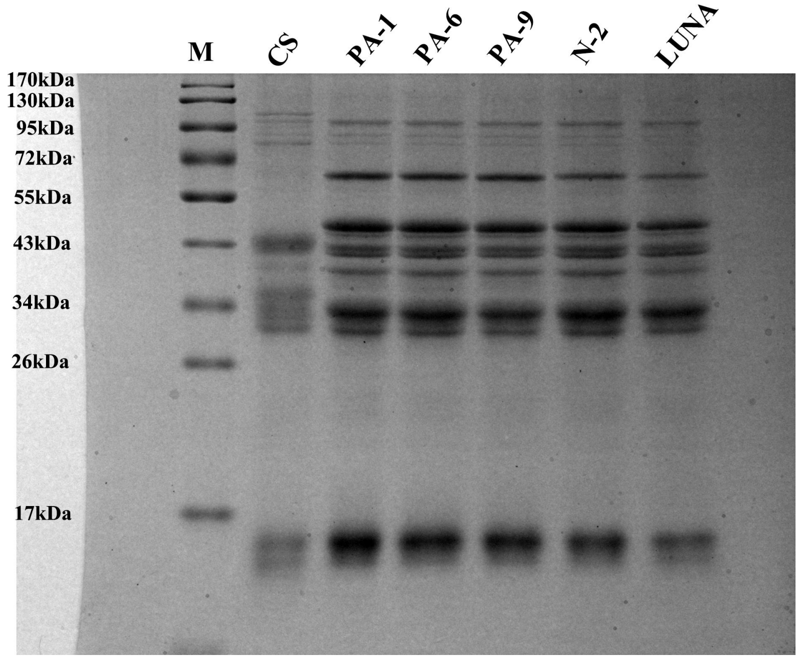

Figure S6. Original SDS-PAGE of PINA distribution in soluble gluten protein fractions extracted from flour (Which appeared in Figure S1 A lower panel (Left).

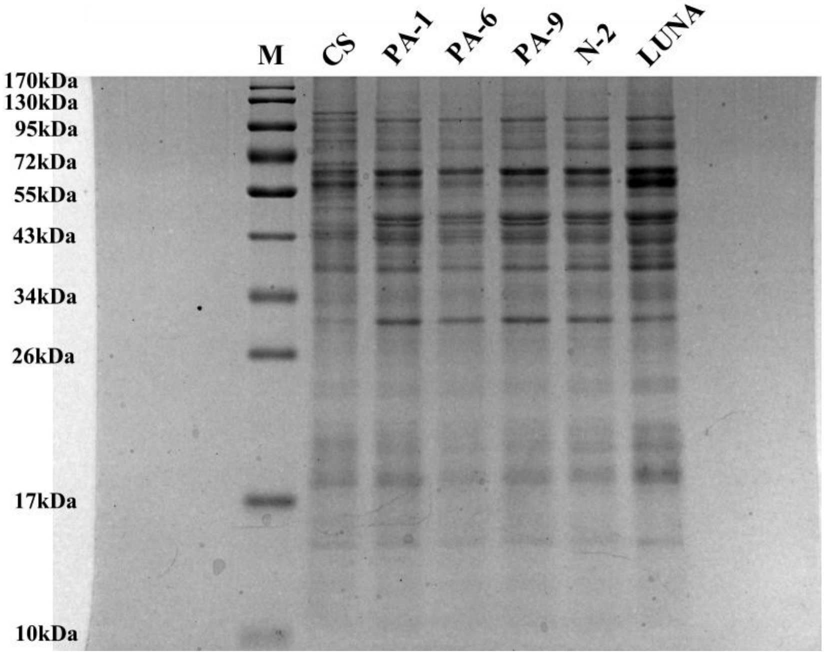

Figure S7. Original SDS-PAGE of PINA distribution in insoluble gluten protein fractions extracted from flour (Which appeared in Figure S1 A lower panel (Right).
